# Fabry–Perot Interferometer Used to Measure Very Low Static Pressure Measurements

**DOI:** 10.3390/s23146493

**Published:** 2023-07-18

**Authors:** Sergio Calixto, Roberto Zitzumbo, Zacarías Malacara Hernandez

**Affiliations:** 1 Centro de Investigaciones en Óptica, Loma del Bosque 115, Leon 37150, Mexico; zmalcar@cio.mx; 2 Centro de Innovación Aplicada en Tecnologías Competitivas, Omega 201, Col. Industrial Delta, Leon 37545, Mexico; rzitzumb@ciatec.mx

**Keywords:** sensor devices, pressure sensor, physical sensor, optical sensor, Fabry–Perot interferometer, polydimethyl siloxane, very low pressure measurements, high sensitivity

## Abstract

This paper describes the use of an optical instrument, the Fabry–Perot interferometer, adapted to measure very low pressures. The interferometer consists of two high-reflectance flat mirrors placed one in front of another. In addition, a metallic chamber contains air or a gas. In one of the faces of the chamber, a flexible thin silicone membrane is attached and, over it, one of the mirrors is glued. The other mirror rests in a fixed mechanical mounting. Light crosses both mirrors and, when it leaves them, forms an interference pattern consisting of concentric circular fringes. When the pressure is increased/decreased within the chamber, a displacement of the fringes is observed due to the movement of the glued mirror. By measuring the fringe displacement and knowing the pressure, a calibration plot can be made. Minimum pressure measurements of about tens of Pascals were achieved.

## 1. Introduction

Pressure [1,2] is defined as the amount of force exerted by a gas or liquid applied to a unit area. Pressure sensors designed to measure the physical pressure of gases and liquids can be classified into several types, like aneroid barometer, manometer, bourdon tube, vacuum (Pirani) and piezoelectric, to mention but a few. Pressure sensors are built on different technologies, design, performance, stability and cost.

Some points to consider in selecting a pressure sensor are: sensor type, operating pressure range, process conditions of application, output type, accuracy, resolution, operating temperature and more. The use of pressure sensors finds a wide range of applications in industry, medicine, semiconductor processing, environmental applications, the manufacturing process, automotive and more. We next describe some pressure instruments based on membranes and diaphragms.

Tran et al. [3] described a piezoresistive n-type silicon pressure sensor for low pressures. It is made with MEMS technology, a combined cross-beam membrane and a peninsula (CBMP) is presented. These sensors consist of N-type piezoresistors arranged in a Wheatstone bridge configuration. The pressure load applied to the membrane produces deformation. The pressure measuring range goes from 0.5 kPa to 5 kPa. Unfortunately, some drawbacks are presented when using these types of sensors. Silicon wafers and photolithography are used with five masks in the fabrication. Piezoresistors are formed via boron implantation. In addition, difficulties associated with nonlinearity, chip size, high costs and applications in industrial fabrication processes remain. Also, because it is an electronic device, electromagnetic fields affect its performance.

Among the systems dedicated to measuring pressure are optical devices that use a membrane. A description of several examples of these are described in references [4,5]. The first one works on a polydimethyl siloxane (PDMS) pad with 8 mm thickness. Two plastic PMMA fibers are attached to it. One of the fibers crosses perpendicularly on top of another identical fiber to create a single pressure point at the intersection of both fibers. Pressure applied to the PDMS pad produces mechanical deformation. This changes the amount of light traveling through the optical fiber. Light deflects when the fiber suffers bending or pronounced curving. Consequently, some energy is lost due to light dispersion. The amount of light conducted by the fiber decreases due to the sensor’s curvature bending. A photodetector measures the change in the light intensity, and from it, calculates the exerted pressure on the diaphragm. The nonlinear response of the sensor begins to show small linearity changes around 63 kPa to 127 kPa, while there is a great change from 127 kPa to 509 kPa. The photodetector sensitivity is higher in the higher-pressure range. Unfortunately, this pressure system shows some drawbacks. Very good PMMA fibers should be used because cheaper ones show core and cladding diameter variation from length to length, creating an imperfect contact point between the fibers that prevents them from properly deforming when pressed. Also, the PMMA fibers show strong hardness, which prevents the system from measuring very low pressures. In its very low range, it presents very low sensitivity. Another drawback is the coupling losses (>30 dB) presented between the LED light source and the photodiode detectors. The current generated is very small 1 μA. Thus, costly and very good instrumentation should be used.

Calixto et al. [5] described a second optical pressure sensor. It consists of a thin flat silicone membrane attached to a chamber filled with water. A light source is produced by means of an optical fiber. When pressure in the chamber is increased/decreased, the membrane produces a convex/concave deflection behaving as a lens. At the output of the lens, a fiber optic collects the refracted light, which increases its intensity when the lens behaves as a positive lens or decreases when the lens behaves as a negative lens. The pressure varied between 3.45 kPa to 20 kPa. Although this device is immune to electromagnetic forces, the light intensity output of the source should be kept stable or fixed.

The third pressure optical system has a flexible positive lens [6]. Here, the lens is attached to one side of a chamber, and on the other side, a flat glass plate closes the chamber. The lens forms an image of an object illuminated with white light on the focal plane. When the pressure increases, the image blurs. A calibration plot is built by measuring the image visibility as a function of the chamber pressure. Hence, the pressure measurement is calculated by measuring the visibility of the image. The pressure range for this sensor lies between 0.69 kPa and 34 kPa. 

Among industrial low-pressure sensors are those shown in references [7,8]. The first one is a tonometer, used in ophthalmology to measure the eye’s intra-ocular pressure (IOP). There are different types of tonometers, like applanation, non-contact, indentation, rebound and Pascal. Their measuring ranges are between 10 and 21 mmHg (1.33 kPa and 2.8 kPa). Unfortunately, these devices are focused to measure the IOP only.

The second one [8] is based on optical fibers and is used in cardiovascular, pharmacology, neuroscience (intracranial pressure), urology, ophthalmology, arterial blood and respiratory fields. The pressure range is between −300 mmHg and +300 mmHg (−40 kPa to +40 kPa). Unfortunately, these sensors should use a light source that emits many wavelengths, which makes them expensive. 

Here, in this manuscript, we show a new kind of optical pressure sensor that uses a Fabry–Perot interferometer. It is proposed as having a low cost, high sensitivity, does not require expensive equipment, does not require a multistep process, it is not sophisticated or time consuming and does not involve health hazards. The sensor comprises a chamber that has, on one end, a thin transparent flexible ring membrane with a high-reflectance mirror glued to it. When the pressure increases/decreases in the chamber, the mirror will suffer a displacement. To form the optical Fabry–Perot cavity, another mirror is placed in front of the first one at some distance. After the light has crossed the cavity, an interference pattern is shown, consisting of a series of concentric light rings, which will move accordingly to the pressure change. To have a calibration curve, the independent variable, the pressure, is measured. The dependent variable is the light fringe displacement, which, by means of a reticle (scale), is measured. This method can give pressure measurements in the order of tens of Pascals. 

## 2. Fabry–Perot Interferometer

**Figure 1:**
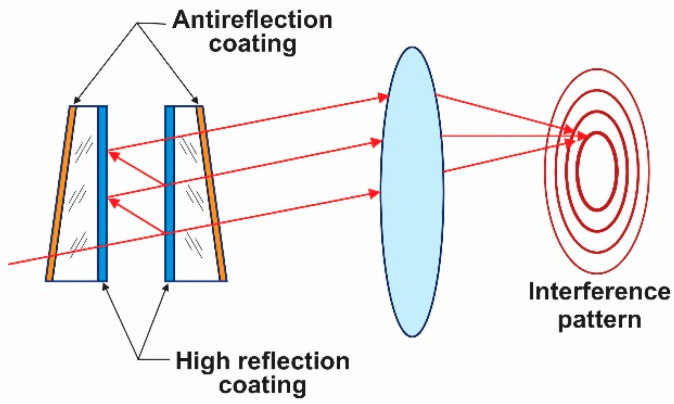
FP configuration.

Figure 1 shows the basic optical configuration of an FP, as described elsewhere [9,10]. Two flat mirrors with a high-reflectance thin film stack in their interior surfaces form an optical cavity, where the light is reflected many times between the thin-film stacks. After the light traverses the FP, a lens focuses light at its focal distance, giving an interference pattern consisting of a series of concentric rings.

The equation [10] that describes the FP interference pattern is:

(1) $$I_{t}=\frac{I_{0}}{\left(1+\left(\frac{4r^{2}}{{\left(1-r^{2}\right)}^{2}}\right){sin}^{2}\left(\frac{\delta }{2}\right)\right)}$$

**Figure 2:**
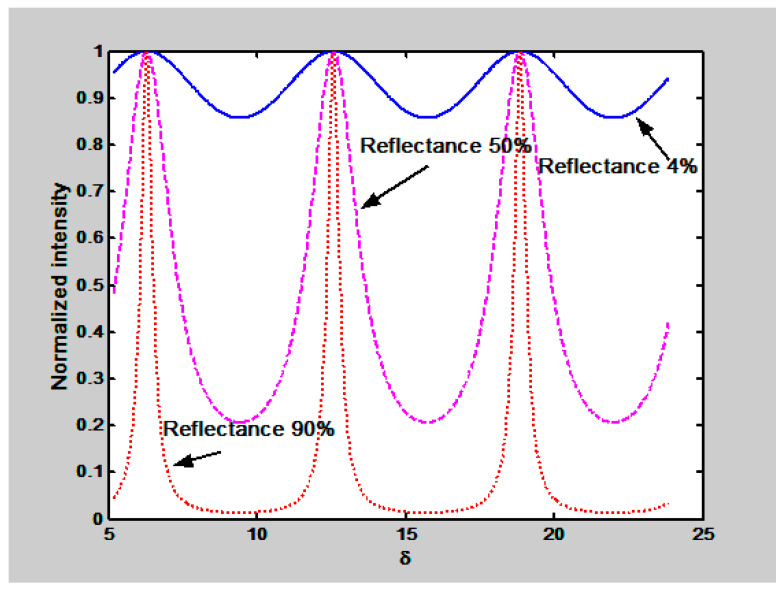
Behavior of interference fringes as a function of δ. Mirror reflectance is the parameter.

Here, *δ =* (*4π*/*λ*) *ns cos ϕ*, s is the spacing between the inner faces of the mirrors, *n* is the refractive index of the material between the mirrors, *r*^2^ is the reflectance and *λ* is the wavelength (*λ* = 632.8 nm for a HeNe laser). We can plot the transmitted intensity as a function of *δ*, with the cavity having a spacing of 0.2 mm, Figure 2. Reflectance values of 0.04, 0.50 and 0.95 were chosen as parameters. We can notice that, as the reflectance of the surfaces is increased, the fringes due to multiple reflections become sharper.

**Figure 3:**
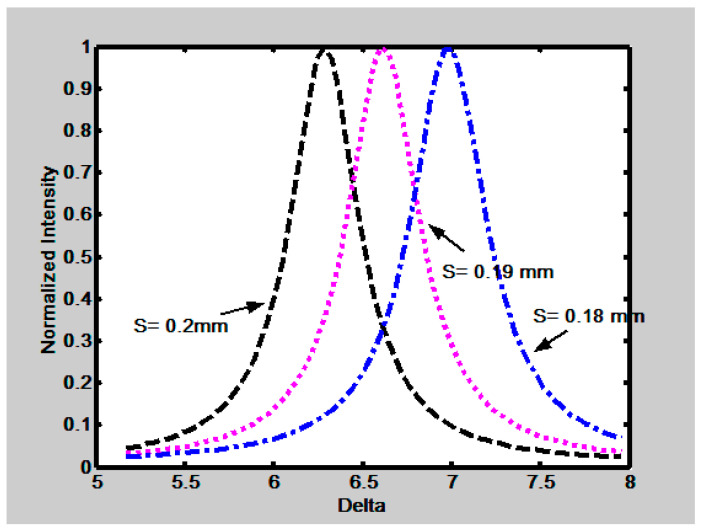
Normalized intensity as a function of δ. The parameter is the spacing “s” between the F-P mirrors. Notice the fringe shifting.

In addition to the behavior presented in Figure 2, it is possible to describe the behavior of the displacement of an interference line; as a function of δ, the mirror spacing *s* is the parameter, Figure 3. The position of the interference line changes with the spacing.

## 3. Membrane Material

The selected material to fabricate the flexible membrane was silicone [11]. This material shows high purity, moisture resistance, thermal stability, flexibility and a non-toxic nature, to mention but a few. 

An uncured translucent silicone rubber compound was chosen with a viscosity of 50,000 cp/mPa.s. A transparent agent was used to cure the silicone rubber through an addition reaction (chain reaction). The curing agent had a viscosity of 550 cp/mPa.s, and both materials were acquired from Dow Corning under the trade names Silastic^®^ T-2 translucent base and Silastic^®^ T-2 curing agent, respectively. All materials were used as received without further purification.

### 3.1. Sample Preparation

In a glass container, to decrease its viscosity, a sample of the silicone rubber was heated to 50 °C in a water bath. Subsequently, the hot silicone rubber and curing agent at room temperature were poured into a kitasato flask at a ratio of 10 parts silicone rubber to 1 part curing agent (10:1).

The kitasato flask was connected to a vacuum pump and sealed with a rubber stopper. The rubber stopper was perforated in the center and a glass rod was adapted to manually stir the blend of components. The vacuum generated inside the kitasato flask helped to extract the air bubbles that formed inside the mixture while the mixing process was carried out.

**Figure 4:**
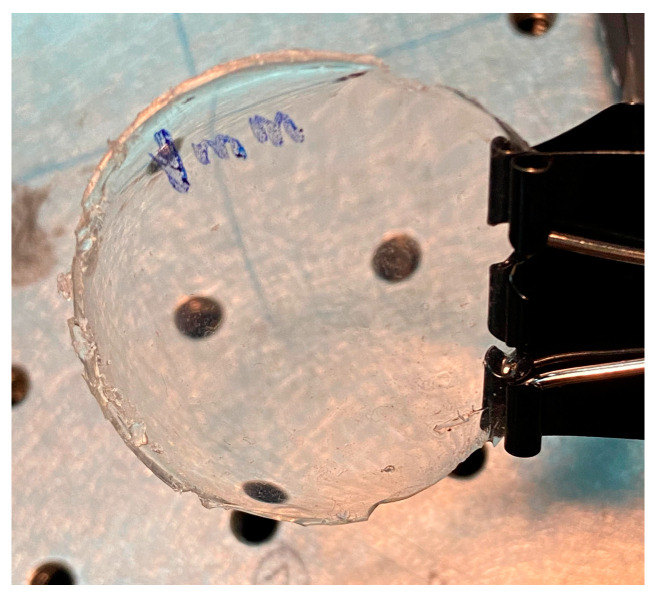
Sample of a silicone membrane 1 mm thickness.

At the end of the blending process, the bubble-free blend was poured onto a leveled glass plate. Then, a film was formed by adjusting the thickness with a scraper. The glass plate was introduced into a heating oven for curing at 50 °C for 45 min. This process allows one to make cured films, ranging from a few hundreds of microns to about 4 mm thickness, Figure 4.

### 3.2. Mechanical Properties

The mechanical properties of the cured silicone rubber samples were determined experimentally in an Instron universal testing machine [12], model 5565. The mechanical properties of the material were measured on bone-type specimens in accordance with the ASTM D-412 standard at a deformation rate of 500 mm/min at room temperature. The resulting mechanical properties of the cured silicone rubber samples are tensile strength 4.7 ± 0.3 MPa, elongation 300 ± 15% and hardness shore A 40 ± 1.

On the other hand, it is known [13] that the air bubbles occluded in the polymeric matrix can retard the initiation process of the chain reaction, since free oxygen radicals can inhibit the reaction of the chemical species present in the reaction medium, in such a way that the mechanical properties of thermosetting materials depend mainly on the nature of their chemical structure, composition and extent of the crosslinking degree, among others [14].

In this context, cured silicone rubber films exhibit excellent optical clarity on unfilled samples and are fully transparent. However, its mechanical properties are poor, mainly those of tensile strength and tear strength. To improve these deficiencies, the literature has reported [15,16] the synthesis of reinforced silicone rubber, which after vulcanization, forms translucent films with good optical clarity and better mechanical properties.

Cured silicone rubber samples exhibit weather, heat, and chemical resistance properties superior to those of other natural and synthetic rubbers [17,18]. It is worth mentioning that, when a rubber undergoes a curing process (vulcanization), a series of chemical reactions occur inside the material, forming a reticulated three-dimensional structural network [19]. The structure formed inside the vulcanized rubber is responsible for providing unique properties, such as flexibility and elasticity with shape memory. That is, it can be deformed under the application of an external force and returns to its original shape when the force ceases. This cycle of elasticity (deformation/elastic recovery) depends mainly on the degree of molecular crosslinking. The silicone rubber’s high sensibility to elastic deformation makes it an ideal material in optical devices to measure low pressures.

## 4. Experimental Optical Configuration Used to Measure Low Pressures

**Figure 5:**
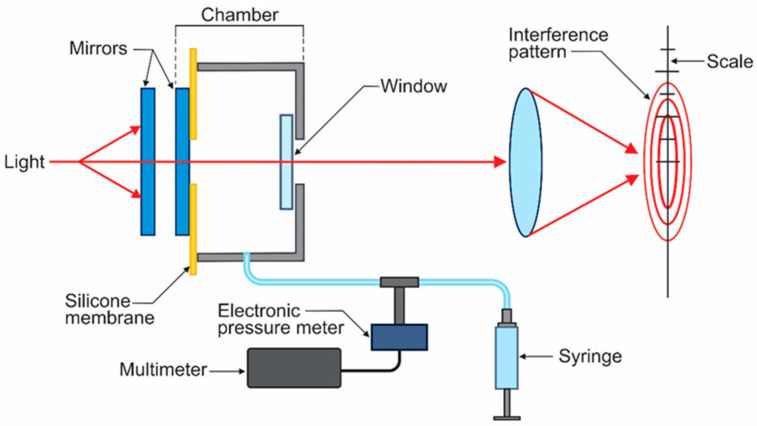
Experimental configuration. A syringe was used to increase the pressure in the chamber. An electronic pressure manometer was used for FP set-up calibration. Lens focused the light forming the interference pattern, which was superposed on a reticle (scale).

**Figure 6:**
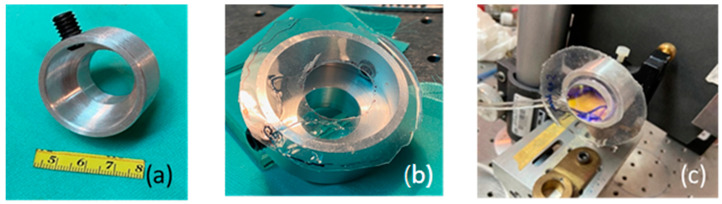
(**a**) Metallic chamber. (**b**) Metallic chamber with ring silicone membrane attached. (**c**) Mirror glued to ring membrane.

**Figure 7:**
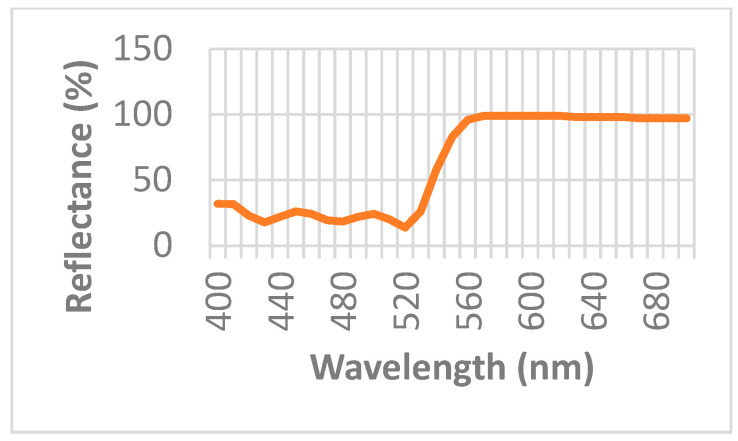
Mirror’s reflectance (%) as a function of wavelength (nm).

**Figure 8:**
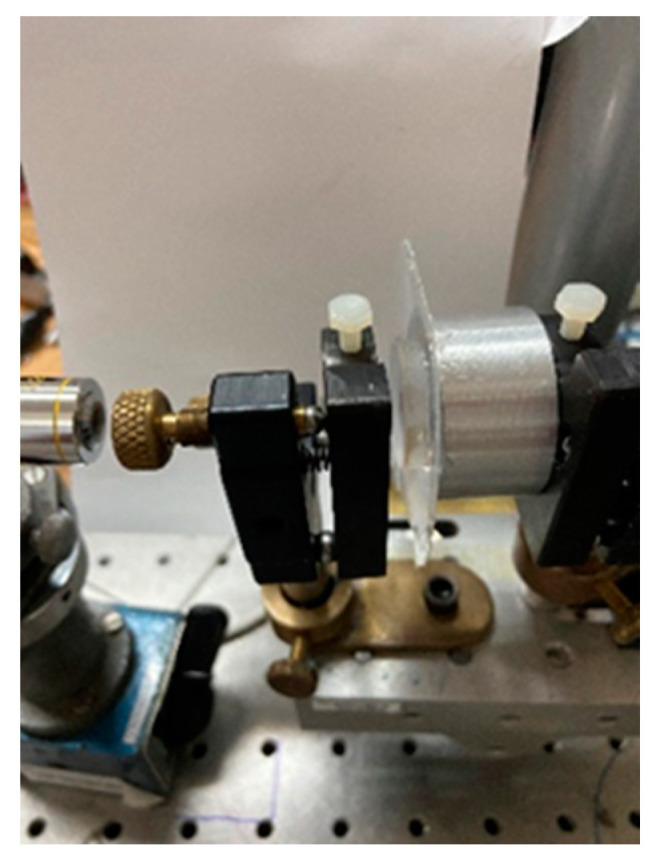
FP interferometer.

In the past, the FP interferometer has been adapted to measure relative humidity [20]. The optical configuration presented there is similar to the one shown in this manuscript. In our case, a constant ambient temperature of 24 °C was maintained during the experiments. A He-Ne laser lighted the interferometer with a wavelength of (λ = 632.8 nm). Figure 5 shows the optical set-up. The main element in the optical configuration is the chamber made in the form of a circular aluminum structure with a diameter of 31 mm and a thickness of 15 mm, Figure 6a. On its front face, a thin ring silicone membrane was glued, Figure 5 and Figure 6b. Different membrane thicknesses were used. To seal the metallic cavity on the back wall of the chamber, a flat glass window was glued. A circular mirror with a diameter of 25 mm was glued to the ring membrane, Figure 6c. The mirror had, on its front surface, a thin-film stack with 99% reflectance, Figure 7; on the back surface, there was an antireflection coating (@ λ = 632.8 nm). A second mirror was placed in a mounting in front of the glued mirror forming the Fabry–Perot cavity, Figure 8. The lens focal distance was 1.2 m. 

The result of increasing/decreasing the pressure in the chamber is the longitudinal movement of the mirror, which changes the spacing between the mirrors. To increase the pressure in the chamber, a syringe was used, which was first connected by means of a hose to an electronic pressure meter [21]. This electronic manometer is uncalibrated from the factory. Thus, a method using a calibrated [22] mechanical manometer was used. After the electronic manometer, a hose connection to the metallic chamber was established.

The method used to calibrate the electronic manometer was next. The mechanical manometer could make measurements between 100 Pa and 3 KPa. A set up was constructed, connecting the mechanical manometer, the electronic manometer and a syringe. Then, the pressure was increased by steps with the syringe and a graph having the independent variable, the pressure and the dependent variable. The voltage given by the electronic manometer was obtained. Then, an equation relating to both variables was obtained. From that equation, it was possible to obtain the pressure values given by the electronic manometer below 100 Pa.

**Figure 9:**
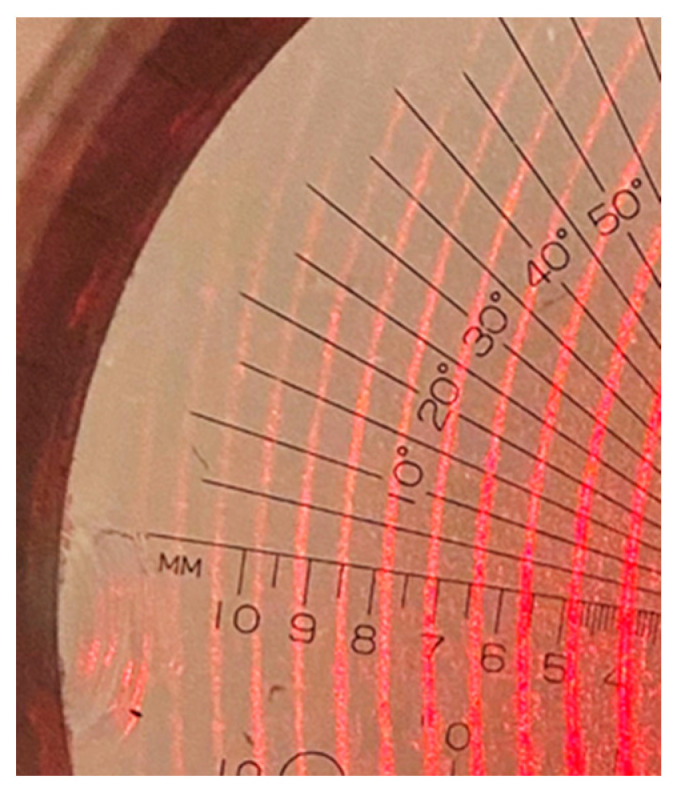
F-P Interference pattern superposed on a reticle. Interference line width in the photograph is thicker than that shown in reality because to take the photograph, a long exposure time was needed to show the reticle. Minimum distance between bars in scale is 100 μm.

After the light left the FP, a lens with a focal length of 1.2 m focused the light, forming the interference pattern superposed on the reticle with a scale, Figure 9. The minimum distance between contiguous scale bars was 100 μm. The lens focal length was chosen to give enough displacement resolution.

When the pressure was increased with the syringe, the interference fringes moved inwards. One fringe was chosen as the pointer, and its movement, as the pressure was increased/decreased, was measured with the scale. Thus, we have the behavior of the distance traveled by the fringe as a function of the pressure. It is noted that for the measurements, the first interference light that is closer to the center of the interference pattern was chosen.

## 5. Results

**Figure 10:**
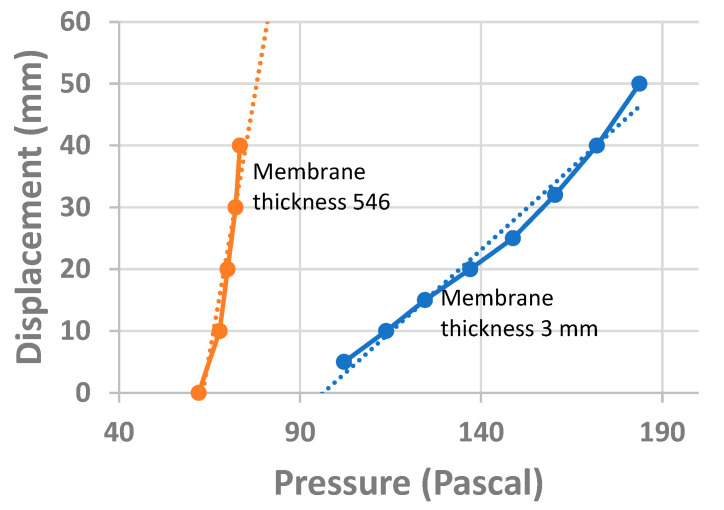
Fringe displacement (mm) vs. pressure (Pascal).

Several experiments were conducted considering parameters, like distance between mirrors and membrane thickness. For the first experiment, two membrane thicknesses were considered, 540 µm and 3 mm. The distance between the mirrors was 1 mm. One light fringe was chosen as the pointer. As pressure increased, the fringe moved, and its displacement was quantified by looking at its position in the reticle. The results can be seen in Figure 10.

A linear interpolation of the plots shown in Figure 10 was performed with the Excel program. The following equations were obtained. For a plot in blue: y = 0.5307x − 51.51 (R^2^ = 0.9806), for a plot in red: y = 3.3577 − 211.95 (0.9133). It is noticed that when the membrane is thicker, more pressure is needed to have fringe displacement; that is, the membrane is stronger. The slope of the plots shows the sensitivity of the optical configuration. The slope of the steepest plot (red color) is given when the thickness of the membrane is 540 μm and it is 3.35 mm/Pascal.

**Figure 11:**
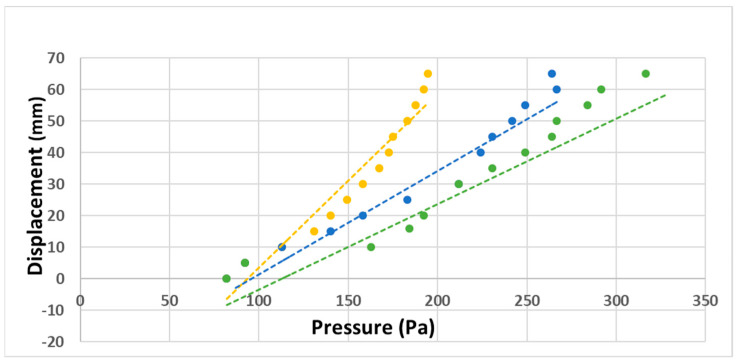
Fringe displacement vs. pressure. Parameter is the distance between mirrors. Distance between mirrors: plot in yellow: 1.5 mm, plot in blue: 2 mm and plot in green: 3 mm.

Another set of experiments that was performed included, as before, the movement or displacement of an interference fringe (dependent variable) as the pressure was increased (independent variable). The parameter was the distance between the mirrors. The membrane thickness was 3 mm. The distances between the mirrors had the following values: 1.5 mm, 2 mm and 3 mm. The results can be seen in the plot shown in Figure 11.

By using the Excel program, the linear interpolated equations for each plot in Figure 11 were calculated, giving the following information: plot in yellow: y = 0.5506x − 51.594 (R^2^ = 0.9338), plot in blue: y = 0.328x − 31.391 (R^2^ = 0.9563) and plot in green: y = 0.2709x − 30.457 (R^2^ = 0.9619). The slopes of the plots are: plot in yellow: 0.55 mm/Pascal, plot in blue: 0.32 mm/Pascal and plot in green: 0.27 mm/Pascal. By looking at the slopes, it is noticed that when the distance between mirrors is smaller, the sensitivity is the greatest, 0.55 mm/Pascal. Thus, there is better sensitivity when the distance between mirrors is small.

## 6. Discussion

The optical configuration that is suggested here is versatile because its sensitivity can be adapted to experimental needs. The parameters to change the sensitivity are: the lens focal distance, the minimum distance between the bars in the scale, the distance between mirrors, mirror’s reflectance and the membrane thickness. The following parameters will increase the sensitivity: long focal distance, short distance between mirrors, small membrane thickness and short distance between bars in the scale. In addition, the use of high-reflectance mirrors will give fringes with a narrow width, which gives the possibility of better displacement measurement. Once the calibration plots have been obtained by measuring the distance traveled by one fringe, it is possible to know the pressure. Another method to “measure” the distance traveled by the fringe would be using a linear CCD. 

Concerning two close pressure measurements, we can take, for example, the plot in Figure 10, series 2. The two upper points in the plot have the coordinates (72.2 Pascal, 30 mm) and (73.4 Pascal, 40 mm). The difference between these points is (73.4 − 72.2 Pa) 1.2 Pascal. Thus, when the light fringe moves 10 mm, a difference in pressure of 1.2 Pascal can be measured.

Table 1 shows the characteristics of some low-pressure methods. However, the method proposed in this manuscript can measure the lowest values of just 10 s of Pascals.

## 7. Conclusions

In this paper, we suggest the combination of flexible PDMS membranes and an FP interferometer to form a sensor that can measure low pressures. Its characteristics make it suitable for industrial, healthcare and biomedical applications.

The technique presented here has some advantages over rigid electronic sensors because it presents: electromagnetic immunity, corrosion resistance, electrical isolation, environmental resistance and high sensitivity. In addition to the fabrication of electronic sensors requiring the use of expensive equipment, they incorporate multistep processes, increasing the probability of obtaining erroneous results and health hazards. 

The flexible FP method that we propose can measure low pressures, is economical and the use of fewer and simpler pieces in the optical configuration means less trouble for a given application. Also, this method can be applied to measure a vacuum with the same principle of measuring the distance that a light fringe moves.

## Figures and Tables

**Table 1:** Table showing the operating pressure range and sensitivity of several methods.

Material or Method	Based on Light or Electronics	Range	Sensitivity	Ref.
Silicon wafer	Piezoresistive element	0–5 kPa	27 mV/kPa	[3]
Optical fibers on PDMS pad	Light	63–509 kPa	−2.5 × 10^−5^ arb units	[4]
Optofluidic, Flexible PDMS flat membrane	Light	3.45 kPa to 20 kPa	0.0513 light intensity/kPa	[5]
Fexible PDMS lens	Light	0.69 kPa to 34 kPa	−0.2 Visibility/kPa	[6]
Tonometer		1.33 kPa to 2.8 kPa		[7]
Fiber optics	Light	−40 kPa to +40 kPa		[8]
Fabry–Perot + Flexible membrane	Light	64 Pa to 323 Pa	3.35 mm/Pascal	This work

## Data Availability

No data were generated or analyzed in the presented research.
